# Metagenomic Insights Into the Effects of Rare-Earth Elements Supplementation on Rumen Digestibility and Meat Quality of Beef Cattle

**DOI:** 10.3389/fmicb.2020.01933

**Published:** 2020-09-29

**Authors:** Lanjiao Xu, Luhua Wen, Yu Ge, Gen Wan, Mingren Qu, Fuguang Xue

**Affiliations:** Jiangxi Province Key Laboratory of Animal Nutrition/Engineering Research Center of Feed Development, Jiangxi Agricultural University, Nanchang, China

**Keywords:** rare earth elements, beef cattle, rumen digestibility, meat quality, rumen metagenome

## Abstract

Rare-earth elements (REE), supplemented as feed additives, effectively improved feed conversion and production performances of monogastrics. However, very little information exists on how REE supplementation affects ruminants. In the present study, twenty-four 18-month-old Jinjiang bull cattle, with initial body weight (BW) of 374.75 ± 14.02 kg, were randomly allotted into four dietary treatments with a 15-day-long preliminary trial: a control treatment (basal diet), a 400 mg/kg REE treatment (basal diet supplemented with 400 mg REE/kg DMI), an 800 mg/kg REE treatment (basal diet supplemented with 800 mg REE/kg DMI), and a 1,200 mg/kg REE treatment (basal diet supplemented with 1,200 mg REE/kg DMI). Based on the results, the optimum supplementation scale was chosen for a 60-day-long follow-up feeding procedure. At the end of the feeding period, all bull cattle were slaughtered. Feed intake, average daily weight gain, carcass performances, meat quality, and rumen microbiota were measured. Results indicate a positive response in terms of growth performance and gastrointestinal digestibility to REE supplementation, and 400 mg/kg DMI treatment presented the most average daily feed intake (ADFI), the best average daily weight gain (ADG), and the least F/G. REE also significantly decreased the ruminal propionate content compared with control treatment. As to microbiota, despite no increases in bacterial community abundance, there was a proliferation of *Bacteroidetes and Tenericutes* and suppression of *Actinobacteria* under REE treatment. Furthermore, REE treatment significantly increased the meat protein content and decreased meat fat content. There was also an increase in the activities of the enzymes related to lipid syntheses. Fatty acid synthetase (FAS) and malate dehydrogenase (MDH) were significantly suppressed, while the activity of the lipolysis-related enzyme, lipoproteinesterase (LPL), was enhanced. In summary, REE supplementation provided an effective regulation on ruminal microbiota, facilitation of ruminal fiber digestibility, promotion of feed conversion, suppression of lipid deposition, and finally, improved the production and meat quality of beef cattle.

## Introduction

Rare earth elements (REE), which comprise lanthanoids, are the fifteenth most abundant component of the earth’s crust (Weber and Reisman, 2012). REE have been shown to effectively improve body weight gain (BWG) and feed conversion (FC) in pigs (He and Rambeck, 2000) and broilers (Redling, 2006), and increase egg production in layer hens (Bandoniene et al., 2018) with a small amount of supplementation (Rim, 2016). Furthermore, studies have indicated that REE, when used as a feed additive for animals, seemed to be safer for humans, for whom the reported acceptable daily intake (ADI) of REE is 0.05–2 mg/kg body weight (Chen and Zhu, 2006). REE might also be a suitable antibiotic alternative in the future husbandry production, for the REE residue in meat is usually lower than that in vegetables and usually does not damage the environment (Wu et al., 2002). However, not much is known about the effects of REE supplementation on the production performance and meat quality in ruminants.

Initial studies of REE have largely focused on its natural marker application to evaluate the ruminal digestibility and the nutrient retention time in the rumen (Keulen and Young, 1977; Crooker, 1982). Furthermore, Yang et al. (2009) showed an effective promotion of REE supplementation on the ruminal digestibility of dietary fiber *in vitro*. However, the effects of REE on ruminant production are still not clear, partially because of the ruminal microbial ecosystem, which is a large functional systematic microbial ecosystem that converts plant material into absorbable nutrients while providing sufficient energy for ruminants (Hobson and Stewart, 2012). REE might exert functions within the gastrointestinal tract, interact with the bacterial micro-flora, and finally, regulate nutrient uptake, digestibility, and utilization (He et al., 2010; Kraatz et al., 2010). Therefore, it is hypothesized that REE supplementation might interact with ruminal bacteria, improve the ruminal digestibility, and promote the production and meat quality of beef cattle.

Metagenomic sequencing methods have been widely applied in detecting the diversity and functions of the gastrointestinal microbiota, and these findings provide us with a deeper understanding of the micro-ecosystem (Pan et al., 2017; Xue et al., 2019a). In this study, the metagenomic sequencing method was chosen to investigate the effects of REE supplementation on rumen microbiota and its potential mechanism on ruminal fermentation parameters, feed conversion, and the nutrient digestibility of beef cattle.

## Materials and Methods

### Animal Preparation, Experimental Design, and Dietary Treatments

Animal care and procedures followed the Chinese guidelines for animal welfare and were approved by the Animal Care and Use Committee of the Jiangxi Agricultural University, and the animal ethic No. JXAULL-20190626. REE was provided in citrate formation (ReC_6_O_7_H_8_ ⋅ 3H_2_O ≥ 99%, Cd ≤ 0.0001%, Pb ≤ 0.002%, As ≤ 0.0005%) by Jiangxi KuangLu technology Co., Ltd. (Jiangxi, China).

Before the main trial, a baseline study was conducted first to investigate the optimum supplementation of REE. Twenty-four 18-month-old Jinjiang bull cattle with average initial body weight (BW) of 374.75 ± 14.02 kg were randomly allotted into four dietary treatments with a 15-day-long preliminary feeding (Watson et al., 2013). Treatments included a control treatment (basal diets without REE supplement), a 400 mg/kg REE supplement treatment (basal diet plus 400 mg REE/kg DMI), an 800 mg/kg REE supplement treatment (basal diet supplemented with 800 mg REE/kg DMI), and a 1,200 mg/kg REE supplement treatment (basal diet supplemented with 1,200 mg REE/kg DMI). Each treatment was repeated 3 times, and two cattle were used for each repeat. The ingredients and nutrient level of the diet are shown in Table 1.

**TABLE 1:** Ingredients and nutrient composition of experimental diets (DM basis).

Items	Proportion
**Ingredients (% of DM)**	
Corn	47.05
Soyabean	8.60
Lime powder	0.5
NaCl	0.35
NaHCO_3_	0.50
Brewers grain	25.00
Straw	15.00
Premix➀	3.00
Total	100.00
**Nutrient composition**	
DM (%)	91.86
NEmf➁/ (MJ/kg)	5.64
CP (%)	11.13
NDF (%)	30.09
ADF (%)	17.63
Ether extract (%)	4.61
Ash (%)	5.04
Calcium (%)	0.62
Phosphorus (%)	0.43

*➀ Ingredients of Premix (per kg): V_*A*_ 350000IU; V_*D3*_ 125000IU; V_*E*_ 3500IU; Fe 3,100 mg; Mn 2,500 mg; Zn 2,200 mg; Cu 720 mg; I 25 mg; Se 15 mg; Co 10 mg. ➁ Calculated value.*

### Digestibility Measurement

The feed was provided artificially for each cattle twice per day at 8:00 and 18:00, and the supply quantity was weighed before feeding. The feed intake of each bull was recorded the next day before 8:00 by weighing the remaining feed. Average daily feed intake (ADFI) was calculated using the following equation: ADFI (kg/d)=∑(a-b)/15, where “a” represented feeding quantity, “b” represents the remaining quantity, and 15 refers to the 15 days of the study.

During the last 3 days, one calf from each repeat with similar body weight was chosen for digestibility measurement, and fecal samples were collected at intervals of 6 h (12 samples in total). Fecal samples were pooled for each calf, dried at 65°C and stored for dry matter (DM), organic matter (OM), Crude protein (CP), neutral detergent fiber (NDF), and acid detergent fiber (ADF) analysis. Fecal excretion and total tract apparent digestibility of dietary nutrients were calculated based on the concentrations of acid-insoluble ash (AIA) in feed and feces (Keulen and Young, 1977). DM was analyzed according to the AOAC, 1990; (method 930.15), while OM was measured through the equation, OM% (DM basis) = 100-ash. Crude protein content was measured according to Chalupa et al. (1991), and NDF and ADF contents were analyzed according to Van Soest et al. (1991). A 5-g sample was placed in 100 ml of neutral detergent (ND) with 50 pL of heat stable amylase (dietary fiber kit; Sigma catalog Number A3306) added before placing the container on heat. Ankom 200 Fiber Analyzer (Ankom Technology, Fairport, NY, United States) was used for the analysis of NDF content. sequential treatment with permanganate was conducted for the ADF measurement.

### Main Trial Feeding Process

Based on the results of the baseline study, the optimum supplementation (400 mg/kg REE supplementation) was chosen for a further 60-day-long feeding procedure (Montgomery, 2007; Thomas et al., 2017). Calves in the control treatment and 400 mg/kg REE supplement treatment were continuously recruited in the main trial. All cattle were raised in the same stall. Each treatment was repeated 3 times, with 2 calves in each repeat. Diets were given to the cattle twice per day at 08:00 and 17:00 and offered a total of 7–8 kg of feed (DM basis) for each calf to meet the nutritional requirement based on the preliminary results. Freshwater was available for *ad libitum* consumption throughout the study.

### Sample Collection and Parameter Measurements

#### Ruminal Fermentation

At the end of the main trial, beef cattle were slaughtered to investigate the effects of REE on meat quality and ruminal fermentable parameters. Ruminal fluid samples from the upper, lower, left, right, and center positions of the rumen were collected immediately after the slaughtering of each cattle. Rumen fluid was uniformly filtered through four layers of sterile gauze and then divided into two portions. One portion was added of metaphosphoric acid, one-tenth volume of the rumen fluid, to subsequently analyze the pH value, VFAs, and ammonia-N (NH_3_-N). The other portion was dispensed into 3 tubes of non-enzymatically sterilized cryotubes, quickly frozen in liquid nitrogen, and then stored at −80°C for analysis of rumen microbiota.

The pH of each rumen fluid sample was measured immediately using a portable pH meter (Testo 205, Testo AG, Lenzkirch, Germany). Individual and total VFAs (TVFA) in the aliquots were measured using a gas chromatograph (GC-2010, Shimadzu, Kyoto, Japan). Ruminal fluid was firstly acidified with 250 μl metaphosphoric acid, one-tenth volume of rumen fluid, centrifuged with 3,000 rpm, and injected directly on to a column containing a porous aromatic polymer (Chromosorb 101). The sample was maintained at 200°C in a gas chromatograph and fitted with a flame ionization detector (Fussell and Mccalley, 1987). The concentration of NH_3_-N was determined by the indophenol method (Terry et al., 2018; Xue et al., 2019b). A standard curve of NH_3_-N content was first created by using standard NH_4_Cl. The absorbance was adjusted through pure water without NH_3_-N content at the 700 nm wavelength. Finally, the absorbance value was measured through the UV-2600 ultraviolet spectrophotometer (Tianmei Ltd., China).

#### Carcass Performances

Carcass weight was weighed using a platform balance (A12-E, Foshan, Guangdong Province, China). Dressing percentage (DP) was calculated by the following equation: DP = (*m*_2_/*m*_1_)×100, where *m*_2_ represented carcass weight, and *m*_1_ represented body weight. The slaughtered beef cattle were acid-discharged at 4°C for 24 h, and the chilled carcasses were then used for analysis (Bryant et al., 1999).

Each carcass was split into halves down the center of the vertebral column from the atlas vertebrae to the caudal vertebrae. Each half was then divided into the shoulder, rib rack, loin, leg, and trim cuts. The backfat thickness and rib sections were split between the 12th and 13th rib using an electric saw. The 12th/13th rib interface was used for measuring backfat thickness (BFT), width, and length of the ribeye using a ruler with a digital planimeter (Pordomingo et al., 2011). The carcass, suet oil, dirty fat, and subcutaneous fat were weighed on an electronic scale balance (Bryant et al., 1999).

#### Meat Quality

Meat pH: The meat pH was measured at 45 min after slaughter and 24 h after acid discharge using the Mettler Toledo Delta 320 pH meter (Mettler Toledo, Greifensee, Switzerland) with a standardized combination electrode and 4.0 and 7.0 buffers for calibration. Measurements at three different positions were taken to determine the pH value, and the mean value was considered as the pH_45min_ and pH_24h_ of each sample (Pordomingo et al., 2011).

Shear force measurement: Shear force was measured based on Wheeler et al. (1996). Briefly, steaks were broiled to an internal temperature 70°C in a thermostat water bath (220 V, 50–60 HZ). Five standard splines were taken from each meat sample along the direction of the muscle fibers, and then cores were sheared with a Warner-Bratzler shear device (Instron Corp., Canton, MA, United States). The mean value was considered as the shear force, which was displayed in kg.

Dripping loss: Three standard meat patches with a size dimension of 2 cm × 3 cm × 5 cm and with a weight of 30 ± 1 g were prepared for each sample and all patches were suspended from fish-hooks in a windless condition for 24 h (Hertog-Meischke et al., 1998). The dripping loss was calculated by the D-value of the initial weight and 24 h weight.

Beef nutrients analysis: The values of DM, ash, meat protein, and intramuscular fat were determined as reported by Andrae et al. (2001). Briefly, 2 grams of meat was weighed on a chemical balance (XS204, ± 0.1 mg;Mettler, Changzhou, China). The meat was dried in an oven (Memmert, Germany) (Table 8) at 105°C for 4 h, and the DM value was calculated by the following equation: *DM*(%) = (*m*_2_/*m*_1_)×100, where *m*_2_ represented drying weight and *m*_1_ represented fresh meat weight. Ash was measured using a Muffle furnace (SX2-15-10; Xinghua Electric furnace factory, Jiangsu, China) at 550°C for 4 h, and the ash content was calculated by the following equation: Ash(%) = (*m*_2_/*m*_1_)×100, where *m*_2_ represented ash weight and *m*_1_ represented DM weight. Meat protein was measured by the Kjeldahl determination method. The distilling apparatus, chemical balance, Kjeldahl flask, and acid burette were applied for the protein content measurement, as described in Pennings et al. (2011). Intramuscular fat content was detected using the Soxhlet extraction method (Steven et al., 2000), and the Soxhlet extraction instruments, ether, and drying oven (Memmert, Germany) were used for the fat content measurement. The fat was measured using the following equation: Fat = (*m*_1_−*m*_2_)/*m*_1_×100, where, *m*_1_ represented weight before ether extraction, *m*_2_ represented weight after ether extraction. A parallel sample was measured for each parameter and results were shown by the mean of each sample.

#### Determination of Fat Metabolism-Related Enzyme Activities

Fat metabolism-related enzymes in the liver were measured to demonstrate the effects of REE supplementation on the dynamics of lipid storage. Activities of fatty acid synthetase (FAS), isocitrate dehydrogenase (ICDH), malate dehydrogenase (MDH), glucose-6-phosphate dehydrogenase (G6PDH), hepatic lipase (HL), lipoprotein esterase (LPL) were determined through the ELISA method. All the kits used here were acquired from Nanjing Jiancheng Bioengineering Ltd. http://www.njjcbio.com.

Based on the manufacturer’s instructions, 0.1 g of liver tissue was separated for each enzyme measurement procedure. For example, for FAS, liver tissue was first mixed with the tissue diluent (5 times volume to the tissue) into homogenate and then centrifuged at 4°C with 3,000 rpm for about 30 min. The supernatant was separated and was allowed to react with coloration liquid on ice for the chromogenic reaction for approximately 20 min. A UV spectrophotometer (Agilent Cary 60) was used for detecting the FAS absorbance at 450 nm wavelength for the FAS content within 10 min after the chromogenic reaction (Clare et al., 2000). Other enzymes were calculated based on the instructions of ELISA kits, similar to FAS. The wavelength in measuring absorbency differed from each other. ICDH, G6PDH, and MDH were measured at 340 nm, while the absorbency of HL and LPL were measured at 710 and 450 nm, respectively. The activity of each enzyme was calculated using the following curve:

$${\mathrm{Activity}}_{n}\,\left(U/g\right)=\left[\left(\Delta \,A_{R}-\Delta \,A_{B}\right)\times V_{t}/\varepsilon_{n}/d\times 10^{6}\right]/V_{S}/T$$

Here, ε*_*n*_* = molar extinction coefficient of the enzyme, *d* = optical path of the cuvette, *V*_*t*_ = total mixed volume, *V*_*S*_ = sample volume, *T* = reaction time, △*A*_*R*_ = absorbency of reaction cuvette, and △*A*_*B*_ = absorbency of the blank.

#### Rumen Microbiota Sequencing Process

DNA for metagenomics sequencing was extracted from the rumen fluid samples by using the QIAamp DNA Stool Mini Kit (Qiagen, Hilden, Germany) according to the manufacturer’s protocols. Briefly stated, 1 ml of rumen fluid was firstly separated and added into a microfuge tube and then mixed with the TE buffers and buffer-saturated phenol respectively (Vaidya et al., 2018). The DNA concentration and purity of the extracted DNA were quantified with TBS-380 (P/N,3800-003, Turner Biosystem, United States) and NanoDrop2000 (Thermo Fisher Scientific, United States), respectively. DNA quality was examined with the 1% agarose gel electrophoresis system.

The 16S rRNA sequencing method was conducted using the barcoded universal primers 338F (5′-barcode-ACTCCTRCGGGAGGCAGCAG-3) and 806R (5′-GGACTACCVGGGTATCTAAT-3′). The primers spanned the V3–V4 hypervariable region so that the effects of REE supplementation on ruminal microbiota can be detected. Illumina HiSeq 4000 platform (Illumina Inc., San Diego, CA, United States) was applied for the sequencing. The QIIME (V1.7.0) quality-control process was performed for the raw tags under specific filtering conditions to obtain the high-quality clean tags and low-quality reads (length < 50 bp or with a quality value <20 or having N bases) were stripped and removed using SeqPrep^1^ (Aronesty, 2013). The effective tags were finally obtained and analyzed by Uparse software (Uparse v7.0.1001). Sequences with >97% similarity were assigned to the same OTUs. The abundance values of OTUs were normalized using a standard of sequence number corresponding to the sample with the least sequences. The GreenGene Database^2^ was used based on the RDP classifier algorithm to annotate taxonomic information (Langille et al., 2013). Alpha diversity and beta diversity were calculated by QIIME (Version 1.7.0)based on this output normalized data and displayed with R software (Version 2.15.3, R Core Team, Vienna, Austria). Principal coordinates analysis (PCoA) was performed by the WGCNA package while cluster analysis was performed by stat packages and ggplot2 package in R software (Version 2.15.3).

### Statistical Analysis

For the digestibility analysis, a normal distribution test was firstly conducted using the SAS (SAS Institute, Inc., Cary, NC, United States) procedure “proc univariate data = test normal” and subsequently, a one-way ANOVA S-N-K test was used to investigate the differences of BWG and digestibility among four treatments. Results were presented as means ± SEM. For the analysis of ruminal fermentation parameter and meat quality, a normal distribution test was also firstly conducted using the SAS procedure “proc univariate data = test normal” and then the student’s *T*-test of SAS 9.2 was applied to analyze the differences between control treatment and REE supplementation treatment. The OTU abundance of ruminal bacteria was first transformed into normal distribution using the log2 transformation, and then the student’s *T*-test of SAS 9.2 was applied to analyze the differences of bacteria. *P*-value <0.05 was considered to be significance and 0.05 ≤ *P* < 0.10 was considered as a tendency. Principal coordinate analysis (PCoA) for different rumen bacteria was conducted using the R package (Version 2.15.3). Spearman correlations between bacteria communities and fermentable and digestibility parameters were assessed using the PROC CORR procedure of SAS 9.2 and then the correlation matrix was created and visualized in a heatmap format using the R package (Version 2.15.3).

## Results

### Growth Performance and Digestibility of Preliminary Trials

Growth performance and digestibility were firstly measured in the present study to investigate the proper supplementation dosage and these results are shown in Table 2. Compared with other treatments, REE supplementation with 400 mg/kg DMI performed the best average daily weight gain (ADG) and feed intake to body weight gain ratio (F/G) among all treatments, and significantly increased the ADG and decreased F/G compared with control treatment. No significant differences were found on the average daily feed intake (ADFI) among all treatments. REE Supplementation with 400 mg/kg DMI and 1,200 mg/kg DMI significantly promoted the digestibility of NDF compared with the control group. No differences were found among the four treatments on the digestibility of DM, OM, CP, and ADF. In summary, the growth performance and digestibility were optimal with 400 mg/kg DMI REE supplement treatment, and animals in this treatment were selected to further investigate the effects of REE on beef cattle.

**TABLE 2:** Effects of REE supplementation on feed conversion and digestibility of nutrients of Jinjiang cattle (%).

Items	REE level (mg/kg)				SEM	*P*-value
	0	400	800	1200		
ADG/kg	0.66^b^	1.01^a^	0.97^a^	0.84^ab^	0.04	0.034
ADFI/kg	5.75	6.21	5.99	5.90	0.10	0.370
F/G	8.21^a^	6.24^b^	6.41^b^	7.13^ab^	0.29	0.049
DM	82.85	84.56	83.73	85.18	0.47	0.359
OM	84.59	87.00	85.90	87.78	0.41	0.058
CP	77.52	78.11	79.32	80.59	0.97	0.747
NDF	62.66^b^	68.64^a^	64.96^ab^	68.87^a^	0.99	0.040
ADF	65.99	66.39	66.25	72.85	1.19	0.096

*ADG, average dairy weight gain; ADFI, average dairy feed intake; F/G, feed intake/body weight gain. DM, dry matter, OM, organic matter, CP, crude protein, NDF, neutral detergent fiber, ADF, acid detergent fiber; SEM, standard error of the mean. a,b means within a row with different letters differed significantly (*P* < 0.05).*

### Growth Performance and Ruminal Fermentation Parameters of the Main Trial

As shown in Table 3, REE supplementation significantly increased the ADG but significantly decreased the F/G ratio (*P* < 0.05). For the ruminal fermentation, REE decreased the ruminal propionate content compared with CON (*P* < 0.05). REE also effectively increased the ruminal total VFAs and acetate, however, the increase was not statistically significant. No changes were found on ruminal pH, butyrate, and NH_3_-N between REE and CON treatments.

**TABLE 3:** Effects of REE supplementation on production performances and ruminal fermentable parameters of Jinjiang cattle.

Items	CON	REE	SE	*P*-value
ADG/kg	0.69^b^	0.83^a^	0.06	0.031
ADFI/kg	6.03	6.38	0.22	0.164
F/G	8.38^a^	6.51^b^	0.46	0.028
pH	7.09	7.14	0.18	0.856
NH_3_-N (mg/dL)	13.62	13.83	1.75	0.427
TVFA (mmol/L)	66.58	69.58	1.83	0.115
Acetate (mmol/L)	42.60	43.60	2.21	0.700
Propionate (mmol/L)	14.03^a^	12.70^b^	0.78	0.030
Butyrate (mmol/L)	10.28	10.95	0.19	0.230
A/P	3.35	3.11	0.72	0.270

*SE, standard error. a,b means within a row with different letters differed significantly (*P* < 0.05).*

### Rumen Microbiota

A total of 6667 OTUs, 17phyla, and more than 300 genera were identified after quality control, and all the taxonomic information is displayed in Supplementary Table 1. All identified bacteria were chosen for further analysis to investigate the effects of REE supplementation on ruminal bacteria.

**FIGURE 1:**
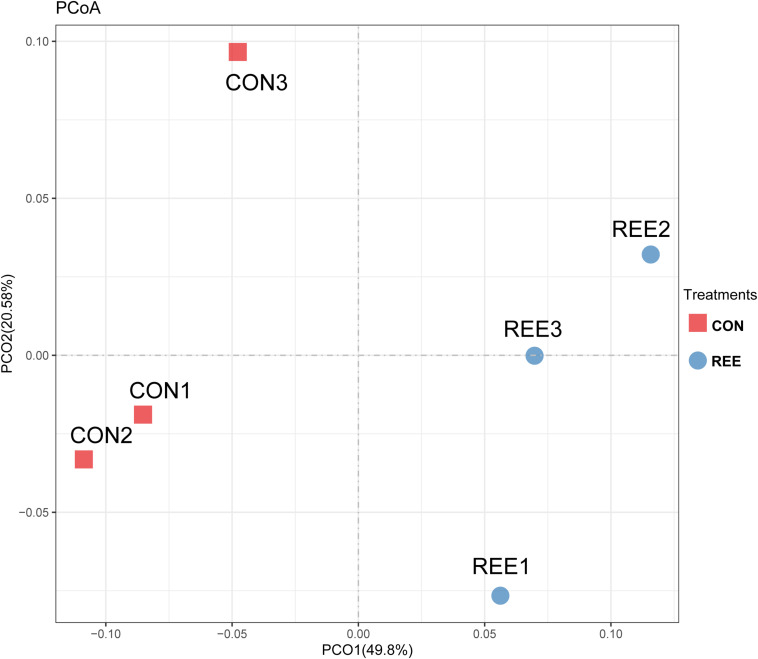
Principal coordinate analysis (PCoA) of bacteria community structures in CON and REE treatment. CON, control diet; REE, rare earth elements.

Alpha diversity was measured by analyzing the complexities of species diversity by running a sample through Chao1, Shannon, Simpson, ACE indexes. The results are shown in Table 4. No significant changes were found between REE treatment and CON. Subsequently, beta diversity analysis was conducted to evaluate the differences of bacterial community between REE and CON. The PCoA result indicated that the PCoA axes 1 and 2 accounted for 49.8 and 20.58 % of the total variation, respectively. The bacterial community in REE treatment could be separated from those in the CON by PCo1 as shown in Figure 1.

**TABLE 4:** Effects of REE supplementation on alpha diversity of ruminal bacteria.

Items	CON	REE	FC	log_2_FC	SE	*P*-value
Ace	4039	3980	0.99	−0.02	0.304	0.854
Chao1	3822	3589	0.94	−0.09	0.126	0.424
Shannon	5.65	5.65	1.00	0.00	0.040	0.988
Simpson	0.012	0.0116	0.97	−0.05	0.005	0.742

*FC, fold change of REE/CON, log_2_FC means logarithmic value of fold change, with the base = 2. SE, standard error.*

Differential analysis on ruminal microbiota at different levels was then conducted. As shown in Table 5, *Bacteroidetes*, *Firmicutes*, *Proteobacteria* contributed the most 3 abundant phyla of ruminal microbiota. REE supplementation significantly promoted the proliferation of *Bacteroidetes and Tenericutes* while suppressing *Actinobacteria*. No significant differences were found in other phyla. Based on the results in Table 6, *Prevotella*, *Succiniclasticum*, *Paraprevotella*, *Bergeyella*, *Tannerella* were the most abundant genera. The abundance of *Bergeyella*, *Fretibacterium*, *Bifidobacterium*, and *Fibrobacter* were significantly increased while the *Anaerovibrio*, *Streptococcus*, and *Selenomonas* populations significantly decreased after REE supplementation compared with CON treatment. No significant differences were detected for other genera.

**TABLE 5:** Effects of REE supplementation on ruminal bacteria abundance (level of phyla).

Items	CON	REE	FC	Log_2_FC	SE	*P*-value
*Bacteroidetes*	56559^b^	71150^a^	1.26	0.33	0.043	0.003
*Firmicutes*	42475	43146	1.02	0.02	0.113	0.82
*Proteobacteria*	2470	2448	0.99	−0.01	0.046	0.865
*Tenericutes*	714^b^	2438^a^	3.41	1.77	0.421	0.038
*Verrucomicrobia*	1392	1207	0.87	−0.21	0.574	0.674
*Actinobacteria*	2243^a^	216^b^	0.10	−3.38	1.160	0.024
*Planctomycetes*	1051	881	0.84	−0.25	0.750	0.849
*Synergistetes*	1195	682	0.57	−0.81	0.420	0.076
*Spirochaetes*	378	316	0.84	−0.26	0.340	0.374
*Fibrobacteres*	83	163	1.97	0.98	0.680	0.174
*Lentisphaerae*	101	66	0.65	−0.63	0.950	0.566
Others	925	664	0.72	−0.48	1.020	0.575

*a,b,means within a row, different letters differed significantly (*P* < 0.05). SE, standard error; FC, fold change of REE/CON, log_2_FC means logarithmic value of fold change, with the base = 2.*

**TABLE 6:** Effects of REE supplementation on ruminal bacteria abundance (level of genera).

Items	CON	REE	FC	log_2_FC	SE	*P*-value
*Prevotella*	33786	27205	0.81	−0.31	0.10	0.176
*Succiniclasticum*	9807	9329	0.95	−0.07	0.16	0.827
*Paraprevotella*	3847	6485	1.69	0.75	0.22	0.159
*Bergeyella*	3385^b^	5633^a^	1.66	0.73	0.18	0.009
*Tannerella*	3269	2959	0.90	−0.14	0.23	0.793
*Oscillibacter*	1662^b^	3272^a^	1.97	0.98	0.29	0.023
*Anaerovibrio*	2850^a^	1419^b^	0.50	−1.01	0.25	0.025
*Schwartzia*	2288	1971	0.86	−0.21	0.16	0.554
*Dethiosulfatibacter*	1942	2273	1.17	0.23	0.12	0.421
*Streptococcus*	3058^a^	1800^b^	0.59	−0.76	0.19	0.003
*Butyrivibrio*	2677	3977	1.49	0.57	0.18	0.115
*Coprococcus*	1529	1943	1.27	0.35	0.30	0.688
*Selenomonas*	2066^a^	706^b^	0.34	−1.55	0.42	0.043
*Lachnospiracea*	1317	1206	0.92	−0.13	0.13	0.650
*Ruminococcus*	1310	859	0.66	−0.61	0.34	0.416
*Clostridium*	1182	973	0.82	−0.28	0.24	0.597
*Fretibacterium*	673^b^	1476^a^	2.19	1.13	0.30	0.026
*Bifidobacterium*	112^b^	1900^a^	16.96	4.08	0.18	0.016
*Saccharofermentans*	563	1175	2.09	1.06	0.31	0.027
*Anaerovorax*	657	895	1.36	0.45	0.21	0.374
*Ornithobacterium*	306^b^	1157^a^	3.79	1.92	0.58	0.032
*Anaeroplasma*	627	736	1.17	0.23	0.15	0.487
*Anaerobacterium*	228	616	2.70	1.43	0.86	0.287
*Acetobacteroides*	143	478	3.35	1.75	0.54	0.185
*Treponema*	214	253	1.19	0.25	0.15	0.494
*Acetatifactor*	89	267	2.99	1.58	0.39	0.091
*Succinivibrio*	230	101	0.44	−1.19	0.45	0.234
*Fibrobacter*	86^b^	178^a^	2.08	1.06	0.31	0.038
*Eubacterium*	57	186	3.26	1.70	0.48	0.082
*Faecalibacterium*	48	85	1.76	0.82	0.43	0.185
*Ruminobacter*	60	41	0.69	−0.54	0.94	0.674
*Lactobacillus*	29	69	2.41	1.27	0.40	0.109
others	3124	3517	1.13	0.17	0.17	0.679

*a, b means within a row, different letters differed significantly (*P* < 0.05). SE, standard error; FC, fold change of REE/CON, log_2_FC means logarithmic value of fold change, with the base = 2.*

**FIGURE 2:**
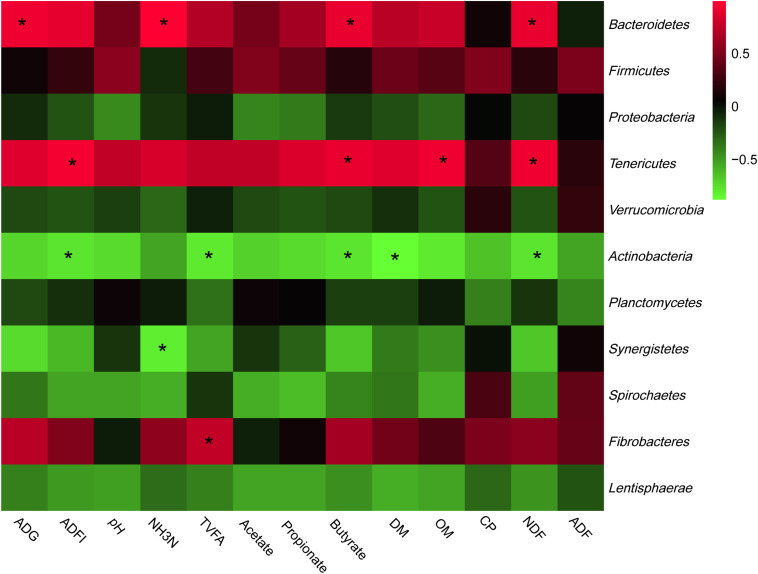
Correlation analyses between the abundance of ruminal bacteria and ruminal digestibility, ruminal fermentation parameters, and growth performances of beef cattle on the level of phyla. The red color represents a positive correlation while the green color represents a negative correlation. “*” means a significant correlation (| *r*| > 0.55, *P* < 0.05).

**FIGURE 3:**
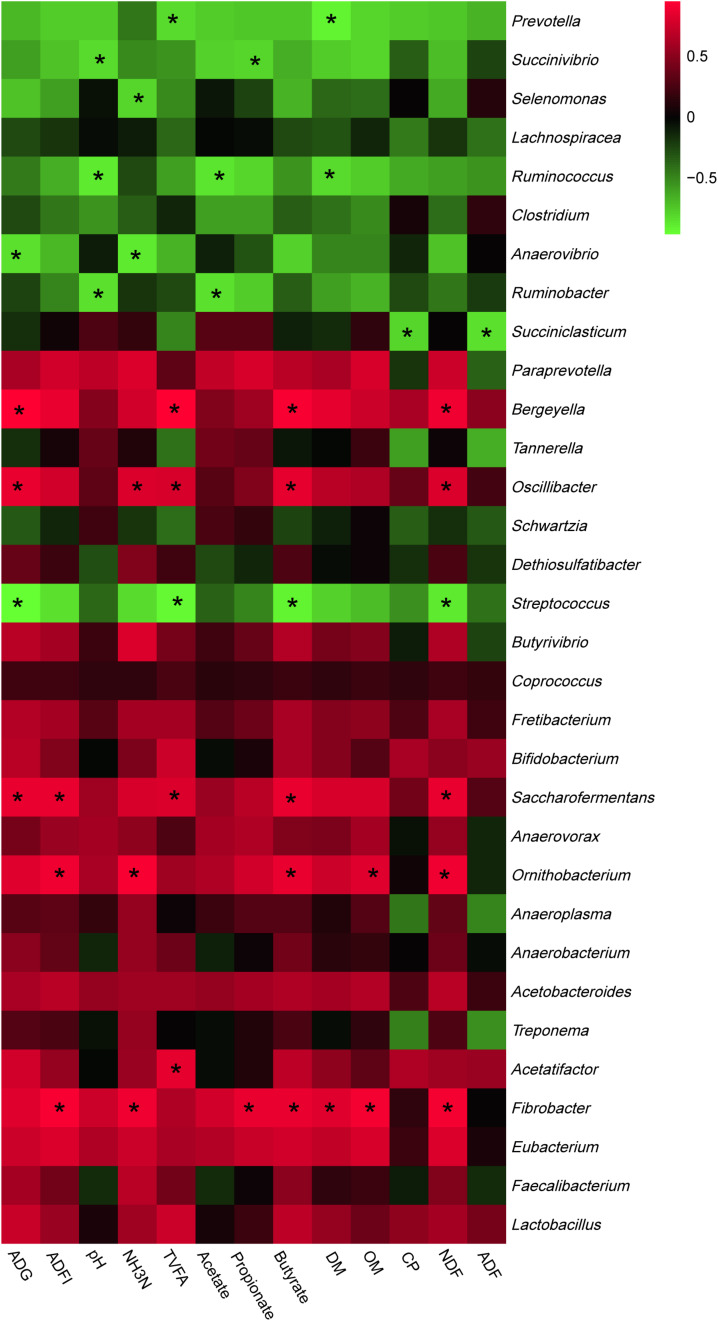
Correlation analyses between the abundance of ruminal bacteria and ruminal digestibility, ruminal fermentation parameters, and growth performances of beef cattle on the level of genera. The red color represents a positive correlation while the green color represents a negative correlation. “*” means a significant correlation (| *r*| > 0.55, *P* < 0.05).

The most abundant phyla and genera were selected for the correlation analysis with the digestibility, ruminal fermentation parameters, and growth performances, and the results are shown in Figures 2, 3, respectively. Based on the results of Figure 2, all phyla could be separated into two clusters. One was positively correlated with digestibility, ruminal pH, and VFAs content, which was mainly consisted of *Bacteroidetes*, *Tenericutes*, and *Fibrobacteres.* The other mainly consisted of *Actinobacteria*, *Synergistetes*, and *Spirochaetes*, which performed a negative correlation with digestibility, ruminal pH, and VFAs content. At the genera level, ruminal microbiota could also be divided into two big clusters. The first cluster mainly consisted of *Prevotella*, *Succinivibrio*, *Streptococcus*, *Succiniclasticum*, and *Ruminococcus*, which showed a positive correlation with digestibility, ruminal pH, and VFAs content. The other cluster showed the inverse correlation and this cluster mainly included *Bifidobacterium*, *saccharofermentans*, *Fibrobacter*, *Acetatifactor*, and *Lactobacillus.*

### Carcass Performance and Meat Quality

Based on the results shown in Table 7, REE supplementation effectively increased body weight and carcass weight. Furthermore, meat protein content was significantly increased while the fat content and DM percentage were significantly decreased after REE supplementation. No significant differences were found on dressing percentage, dripping loss, shear force, and ash content.

**TABLE 7:** Effects of REE supplementation on carcass performance and meat quality parameters.

Items	CON	REE	SE	*P*-value
Body weight	421.6	436.6	6.87	0.147
Carcass	225.9	231.1	8.52	0.574
Dressing percentage	59.55	59.57	1.49	0.993
Dripping loss	0.01	0.01	<0.001	0.810
Shear force	4.95	5.95	0.31	0.063
pH _45min_	6.24	6.22	0.23	0.915
pH _24h_	5.27	5.56	0.31	0.542
Backfat thickness (mm)	14.40	6.22	2.26	0.048
Abdominal fat percentage	7.40^a^	3.47^b^	0.37	0.012
Subcutaneous fat percentage	8.81	6.68	0.67	0.255
Tallow rate	2.78	1.52	0.22	0.039
DM (%)	31.2^a^	29.1^b^	1.40	0.033
Protein (%)	21.2^b^	24.2^a^	1.09	0.024
EE (%)	7.65^a^	4.26^b^	0.93	0.007
Ash (%)	1.81	2.11	0.08	0.060

*DM, dry matter, EE, ether extracts; SE, standard error. a, b means within a row, different letters differed significantly (*P* < 0.05).*

**TABLE 8:** Effects of REE supplementation on activities of lipid metabolism related enzymes in the liver.

Items	CON	REE	SE	*P*-value
FAS (U/mg)	0.59^a^	0.47^b^	0.01	0.047
ICDH (nmol/min ⋅ mg)	9.88	7.67	0.76	0.200
MDH (nmol/min ⋅ mg)	8.68^a^	6.18^b^	0.49	0.022
G6PDH (nmol/min ⋅ mg)	4.91	5.42	0.18	0.385
HL (U/mg)	0.67	0.76	0.14	0.603
LPL (U/mg)	0.93^b^	1.29^a^	0.06	0.015

*FAS, fatty acid synthetase; ICDH, isocitrate dehydrogenase, MDH, Malate dehydrogenase, G6PDH, Glucose-6-phosphate dehydrogenase, HL, Hepatic lipase, LPL, lipoproteinesterase; SE, standard error. a, b means within a row, different letters differed significantly (*P* < 0.05).*

### Activities of Lipid-Metabolism-Related Enzymes in the Liver

Activities of lipid metabolism-related enzymes were measured to investigate the reason for the decrease of fat content. The results are shown in Table 8. REE supplementation effectively suppressed the activities of fatty acid synthetase (FAS) and Malate dehydrogenase (MDH), while improving the activity of lipoproteinesterase (LPL). No significant effects of REE on Hepatic lipase (HL), isocitrate dehydrogenase (ICDH), and Glucose-6-phosphate dehydrogenase (G6PDH) were detected.

## Discussion

### Effects of REE Supplementation on the Ruminal Fermentation and Production Performance of Beef Cattle

The results from the current study showed that the REE supplementation significantly increased the ADFI and ADG of the ruminant, which is in line with the improvement of monogastric animals after REE supplementation (He et al., 2010; Kraatz et al., 2010). The transformation of the ruminal bacteria community, through the enhancement of ruminal fermentation after REE supplementation, might be the causation of the promoted production performances.

High feed efficiency of ruminants was always found coupling with a high abundance of cellulose-degrading bacteria (Li, 2017). In the present study, the main cellulose-degrading bacteria such as *Bacteroidetes* and *Fibrobacter* significantly proliferated after REE supplementation (Spain et al., 2015), which provided a greater degradability on fiber content, especially the NDF, and therefore ADG increased in REE supplement treatment. Also, Andreiadis et al. (2010) demonstrated that the carboxylate binding ability of lanthanide increased through coordinating with gram-negative bacteria, indicating that a greater carbohydrate-binding ability of microbes might be acquired after REE supplementation, and more VFAs and energy would be provided for promoting the growth performance of ruminant. Moreover, REE considerably promotes bacterial secondary metabolism (Yu et al., 2015), which increases nutrient metabolism and absorption in the hindgut. In summary, this study enhanced the production performance of beef cattle.

### Effects of REE Supplementation on Meat Protein

Meat protein content was significantly increased in the REE treatment. This result might indicate that the protein synthetic process in muscle is significantly enhanced through interaction with REE.

Being preliminarily used as a natural marker to evaluate the ruminal digestibility and the nutrient retention time in the rumen (Keulen and Young, 1977; Crooker, 1982), the REE are not degraded greatly in ruminal condition and will pass through the rumen into the hindgut or transport into muscles via ruminal epithelial transporter and blood circulation. Lower concentration of REE has been proved to improve protein synthetic strength and cell activity (Jingchuan et al., 2001), and might further increase meat protein content when transported into muscle tissue. REE was also shown to markedly promote the bacterial cellulose-degrading process (Yu et al., 2015), which improved the microbial activities and provided more nutrients and energy for the protein synthesis process to increase meat protein content.

### Effects of REE Supplementation on Lipid Metabolism

Lipid deposition in both abdomen and meat were significantly suppressed, which reflects the results of a similar study by He et al. (2006). As mentioned above, REE may be transported into the muscle to further modulate lipid synthesis metabolism. Moriwaki and Yamamoto (2013) demonstrated that REE possessed the capacity for lipid-binding and later, that they competitively inhibit lipid synthesis by forming complexes with *N*-acetylglucosamine phosphates, which are the structural components of Lipid A (Ngwenya et al., 2009). Apart from directly impeding the lipid synthetic process, REEs also interact with lipid-metabolism-related enzymes. In the present study, REE significantly reduced the activities of fatty acid synthesis enzymes such as FAS and MDH, and enhanced lipolysis related enzymes such as LPL (Néchaud and Uriel, 1972; Student et al., 1980; Goward and Nicholls, 2008). This result enhanced the lipolysis process but suppressed the adipogenous process, and thus, the lipid deposition in muscle and abdomen was suppressed significantly.

Moreover, interaction with microorganisms that involves the alteration of the ruminal bacteria community and ruminal fermentable metabolites might also down-regulate lipid deposition. In ruminal conditions, lipid metabolism shows a strong correlation with the bacterial biohydrogenation process, which reduces the toxicity of unsaturated lipids for microbial growth (Toral et al., 2018). The biohydrogenation process strengthened after REE supplementation because of the proliferation of *Butyrivibrio* strengthens the biohydrogenation process as one of the most active 18:0-forming bacteria (Jenkins et al., 2008), and the suppression of *Streptococcus Bovis*, which possessed hydrated 18:2n-6 to 13-hydroxy-9-octadecenoic acid, which diverted 18:2n-6 away from the biohydrogenation pathway (Toral et al., 2018). The ruminal saturated lipid content increase was accompanied by an enhanced biohydrogenation process. Since ruminal saturated lipid content might not be utilized by the liver or other organs and afterward excreted with the faces.

Apart from the biohydrogenation process, the *Bacteroidetes/Firmicutes* ratio might also affect lipid deposition. The ratio of *Bacteroidetes*/*Firmicutes* has been shown to affect the ability of nutrient absorbency, and the ratio is strongly correlated with lipid metabolism (Uebanso et al., 2017). The mRNA levels of lipogenic enzymes have also been shown to decrease with the increased ratio of *Bacteroidetes*/*Firmicutes* (Cui et al., 2013). Therefore, the increased ratio of *Bacteroidetes*/*Firmicutes* after REE treatment might partially contribute to the reduction of lipid deposition.

Apart from the ruminal microbial community, rumen microbial fermentation metabolites further impact the lipid composition of meat by providing precursors, the volatile fatty acids (VFAs) for *de novo* FA synthesis in the intramuscular lipid (Shingfield et al., 2013). In the present study, REE significantly decreased propionate content, which proved to play an important role in the synthesis of fatty acids (Tirosh et al., 2019), and thus explained the decrease of lipid content.

In summary, REE supplementation effectively improved the production and meat quality of beef cattle by regulating the rumen microbiota community, facilitating ruminal fiber digestibility, promoting the feed conversion, and suppressing abdominal lipid deposition. The findings of this study suggest a way of increasing meat quantity and the quality of beef cattle production.

## Data Availability Statement

All the raw sequences were submitted to the NCBI Sequence Read Archive (SRA; http://www.ncbi.nlm.nih.gov/Traces/sra/). The datasets for this study can be found under accession number PRJNA604898.

## Ethics Statement

The animal study was reviewed and approved by Animal Care and Use Committee of the Jiangxi Agricultural University.

## Author Contributions

LX designed the study. MQ conducted the experiment. FX analyzed the data and contributed to English editing. LX and FX wrote the manuscript. All authors contributed to the article and approved the submitted version. All authors carefully read and are accountable for all aspects of the work.

## Conflict of Interest

The authors declare that the research was conducted in the absence of any commercial or financial relationships that could be construed as a potential conflict of interest.

## Footnotes

1 https://github.com/jstjohn/SeqPrep

2 http://greengenes.secondgenome.com
